# Destructive Changes in the Neuronal Structure of the FVB/N Mouse Retina

**DOI:** 10.1371/journal.pone.0129719

**Published:** 2015-06-19

**Authors:** Jinnan Yang, ChangLong Nan, Harris Ripps, Wen Shen

**Affiliations:** 1 Department of Biomedical Science, Charles E Schmidt College of Medicine, Florida Atlantic University, Boca Raton, FL 33431, United States of America; 2 Marine Biological Laboratory, Woods Hole 02543, United States of America; Dalhousie University, CANADA

## Abstract

We applied a series of selective antibodies for labeling the various cell types in the mammalian retina. These were used to identify the progressive loss of neurons in the FVB/N mouse, a model of early onset retinal degeneration produced by a mutation in the *pde6b* gene. The immunocytochemical studies, together with electroretinogram (ERG) recordings, enabled us to examine the time course of the degenerative changes that extended from the photoreceptors to the ganglion cells at the proximal end of the retina. Our study indicates that photoreceptors in FVB/N undergo a rapid degeneration within three postnatal weeks, and that there is a concomitant loss of retinal neurons in the inner nuclear layer. Although the loss of rods was detected at an earlier age during which time M- and S-opsin molecules were translocated to the cone nuclei; by 6 months all cones had also degenerated. Neuronal remodeling was also seen in the second-order neurons with horizontal cells sprouting processes proximally and dendritic retraction in rod-driven bipolar cells. Interestingly, the morphology of cone-driven bipolar cells were affected less by the disease process. The cellular structure of inner retinal neurons, i.e., ChAT amacrine cells, ganglion cells, and melanopsin-positive ganglion cells did not exhibit any gross changes of cell densities and appeared to be relatively unaffected by the massive photoreceptor degeneration in the distal retina. However, Muller cell processes began to express GFAP at their endfeet at p14, and it climbed progressively to the cell’s distal ends by 6 months. Our study indicates that FVB/N mouse provides a useful model with which to assess possible intervention strategies to arrest photoreceptor death in related diseases.

## Introduction

Mouse models of photoreceptor degeneration have been investigated for many years in the hope of understanding the causes of photoreceptor death in humans and for the development of therapeutic approaches (cf. Pawlyk et al., 2005) [1]. In addition to the large number of studies dealing with the genetic and biochemical features of photoreceptor degeneration, there have been a number of attempts to characterize the morphological changes of inner retinal neurons, i.e., the remodeling of neurons postsynaptic to the photoreceptor cells undergoing genetically-mediated cell death [2–5]. The purpose of this study was to characterize the sequence of neuronal changes during and after photoreceptor cell degeneration in the FVB/N mouse.

FVB/N is an inbred mouse strain of which the genetic background has been well characterized [6]. These albino mice are homozygous for the *Pde6b* ^*rd1*^ allele encoding the β-subunit of cGMP phosphodiesterase (PDE) [7]. The degeneration is inherited in an autosomal recessive fashion and characterized by a rapid initial loss of rod photoreceptors that is followed by the loss of cone photoreceptors; by postnatal day 35 most photoreceptor cells have degenerated. Interestingly, mutations in the gene encoding the β- subunit of cGMP-PDE have been found in human patients suffering from autosomal recessive retinitis pigmentosa (RPE). Thus, this mouse strain provides an additional model of rapid photoreceptor degeneration, which may relate to the inherited loss of visual cells in human forms of the disease.

## Materials and Methods

### Animals

FVB/N with 129 SvEv background and wild-type 129 SvEv mice were obtained from the Jackson Laboratory (Bar Harbor, ME). The mice were kept in the animal facility under a 12-h dark/light cycle. The illumination levels in the mouse housing room were approximately 200 Lux. The mice used in the experiments were euthanized either by intraperitoneal injection of a mixture of katamin (200mg/kg) and xylazine (10mg/kg) or by cervical dislocation performed on the animals at age of postnatal (p) day 21 and younger. All procedures were performed in accordance with National Institutes of Health guidelines for the care and use of laboratory animals, and were approved by the Committee on Animal Research (IACUC) of Florida Atlantic University.

### Immunocytochemistry

Freshly enucleated eyes were fixed for 20 min in a phosphate buffered saline (PBS) solution containing 4% paraformaldehyde. After removing the cornea and lens, the eyecup remained intact for further processing. It was then placed in the fixative for another 15 min, dehydrated in graded sucrose solutions (10%, 15%, 20% and 30%), and immersed in 30% sucrose overnight at 4°C. The dehydrated eyecups were embedded in OCT compound (Ted Pella, Redding, CA), frozen overnight, and then sectioned at 14μm on a cryostat. Frozen sections were collected on slides, air dried, and stored at -80°C.

For antibody labeling, the sections were rinsed with PBS plus 0.1% Tween (PBST) to which was added 0.3% Triton-X (PBST-T), and then treated with a blocking solution consisting of 10% normal goat or donkey serum in PBST-T. They were then incubated in primary antibodies dissolved in a mixture containing either 3% goat or donkey serum in PBST-T, and held overnight at 4°C (see Table 1 list of primary antibodies, concentration and their sources used in this study). Negative controls were performed with the same solutions, but lacking the primary antibody. After three 15min washes with PBST-T, the sections were incubated in a fluorescent secondary antibody, either Alexa 488 or Cy-3 (Jackson Immunoresearch, West Grove, PA) each at a concentration of 1:1000, for 40 min at room temperature. Sections were subsequently rinsed with PBST, mounted with DAPI in Vectashield mounting medium (Vector Laboratories, Burlingame, CA) and viewed with a confocal laser-scanning microscope (LMS 700, Zeiss, Munich, Germany). Images were acquired with 20x, 40x and 63x oil-immersion objectives, and processed with the Zeiss Microscope Software Zen.

**Table 1:** List of the Antibodies Applied in the Current Study.

Antigen	Antiserum	Working dilution	Source
GFAP	Rabbit anti-GFAP	1:2000	Sigma
PSD-95	Rabbit anti-PSD-95	1:4000	Abcam
M-opsin	Rabbit anti-M-opsin	1:200	Millipore
S-opsin	Rabbit anti-S-opsin	1:200	Millipore
HCN4	Rat anti-HCN4	1:2	F. Müller*
GABA	Rabbit anti-GABA	1:10000	Sigma
Calbindin D28	Rabbit anti-calbindin	1:2000	Sigma
Brn-3a	Mouse anti-Brn-3a	1:500	Chemicon-Upstate
Rohopsin	Mouse anti-rhodopsin	1:1000	Sigma
	Mouse anti-		
PKCα	PKCαMouse anti-	1:500	Sigma
NF200	NF200	1:1000	Millipore
SV2	Mouse anit-SV2	1:10	DSHB
ChAT	Goat anti-ChAT	1:500	Millipore

* Müller et al., 2003.

### Retrograde labeling retinal of ganglion cells

All the procedure of Lucifer Yellow retrograde labeling was performed in a darkroom under a dim red-light illumination. Freshly enucleated eyes with attached optic nerve stumps were dipped into a small drop of a saturated Lucifer Yellow (LY) solution for 20 minutes, then rinsed with Minimal Essential Medium (MEM, Corning Cellgro) to remove the extra dye. The corneas and lenses were removed from the eyeballs and the eyecups were placed in a fresh oxygenated MEM for 30 minutes in room temperature. The eyecups were fixed in a PBS solution containing 4% paraformaldehyde for 30 minutes. The retinas were subsequently dissociated from the fixed eyecups and rinsed several times with a PBS solution. The LY dye retrograde labeled retinas were mounted in the Vectashield mounting medium (Vector Laboratories) and viewed with a confocal laser-scanning microscope.

### Cell counting

The antibody-labeled horizontal cells (HCs), amacrine cells (ACs) and ganglion cells (GCs) in flat-mounted retinas were counted in fields of 320x320 microm and the images were taken from dorsal, ventral, nasal and temporal locations of the retinas. In each location at least 6 images were taken along the radial axis of the retina and a minimum of 24 images were obtained from each retina. The numbers of the cells in control and FVB/N retinas were automatically counted from the images using Image J software (National Institute of Health). The averaged cell number for each result was obtained from 4 to 6 retinas either from the control or FVB/N. The numbers of cells were presented as mean±SEM.

### Electroretinography (ERG)

Mouse ERGs were recorded using protocols modified from those described in an earlier study [8]. Briefly, mice were dark adapted for at least 2 hrs, and anesthetized with a mixture of ketamine (70mg/kg) and xylazine (10mg/kg) injected intraperitoneally. Pupils were dilated with a single drop of 0.5% tropicamide/0.5% phenylephrine hydrochloride, and a heating pad was used to keep the body temperature at 38°C. The corneal electrode was a custom-made Burian-Allen electrode designed for use with mice; a reference electrode was placed in the mouth, and a ground electrode was inserted subcutaneously near the tail. ERG recordings were carried out with mice mounted on an anti-vibration table in a dark, shielded Faraday cage to minimize mechanical and electromagnetic noise. A differential amplifier (Grass Instrument Co.) was used to record ERG responses, which were band-pass filtered between 0.1 and 1000Hz, and digitized at 2kHz with a PowerLab data acquisition device (AD Instruments). To increase the signal to noise ratio, 3–5 light-evoked responses were averaged. A Xenon photostimulator (model PS-22, Grass Instrument Inc., Quincy, MA) delivered 10-msec flashes of white light whose unattenuated irradiance at the cornea was approximately 236 lux/sec (~75.1 cd-s/m^2^), calibrated with a photometer (UDT S370 Optometer, UDT Instruments, CA). For dark-adapted ERGs, the inter-stimulus interval was at least 1min.

The amplitude of the *a*-wave was measured from the pre-stimulus baseline to the *a*-wave trough. The amplitude of the *b*-wave was measured from the trough of the *a*-wave to the peak of the *b*-wave, or if no *a*-wave was present, from the base line to the *b*-wave peak. Implicit times were measured from stimulus onset to the *a*-wave trough or the *b*-wave peak.

## Results

### Early onset loss of photoreceptors

**Fig 1:**
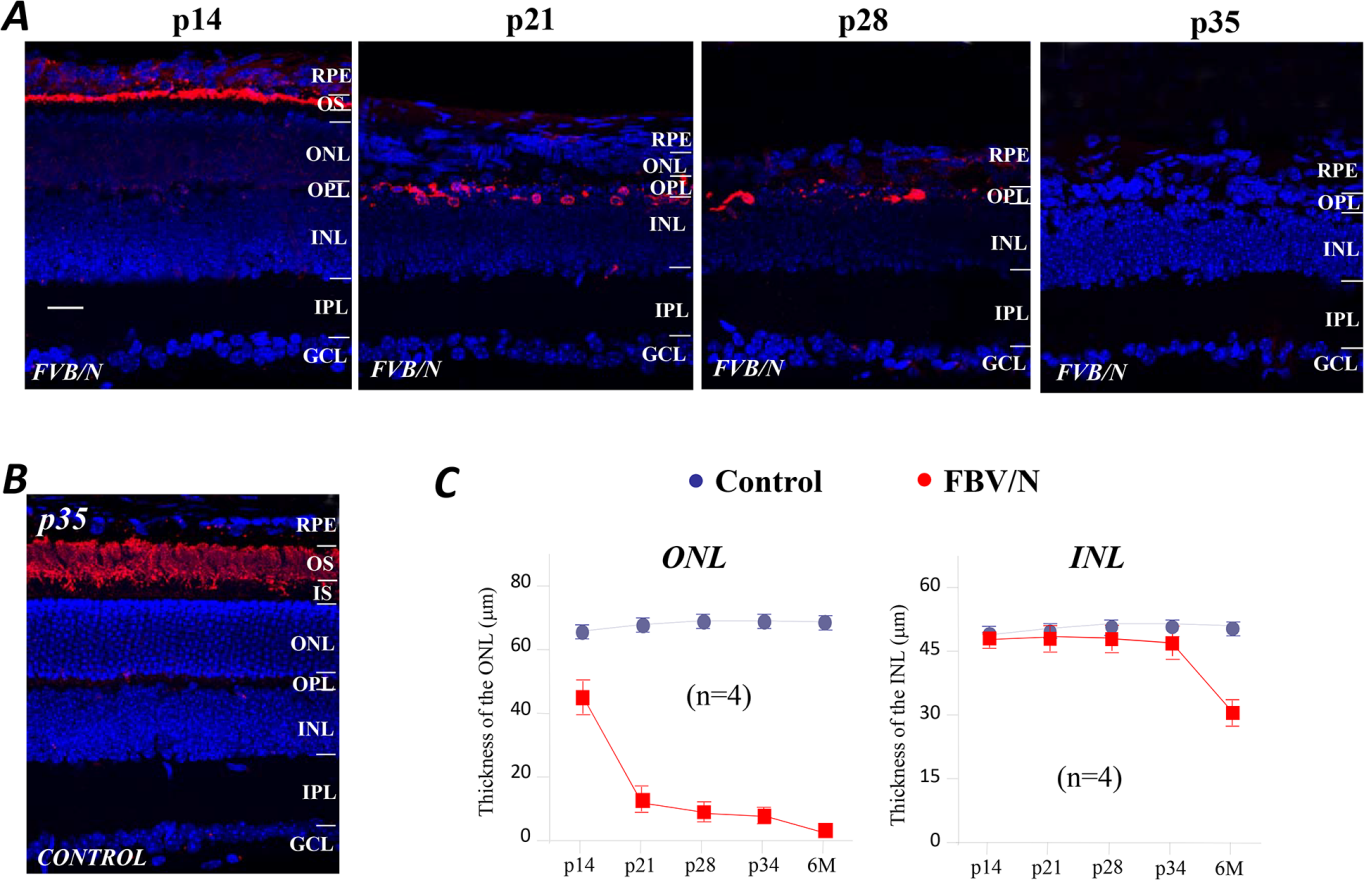
Early onset rod degeneration in the FVB/N mouse. (A) The anti-rhodopsin labeling (red) of rod outer segments and DAPI counterstaining of the cell nuclei (blue) in retinal sections of FVB/N mice at various postnatal ages. By p14 the FVB/N retina has lost a significant number of rods and the retina is thinner than normal, there is a further loss at p21. Apposition of the RPE and the surviving neurons is seen at p28, and at p35 where there is no rhodopsin labeling. (B) Anti-rhodopsin labeling in control retinal sections at the age of p35 displays robust staining throughout the layer of outer segments. (C) The thicknesses of the ONL and INL measured from control and FVB/N retinal sections shows a progressive reduction in FVB/N retinas. Scale bar, 20μm.

Retinal sections prepared from the FVB/N mouse at postnatal (p) day 14, 21, 28 and 35, i.e., from juvenile to early adulthood are shown in Fig 1A together with a section from a control retina at p35 (Fig 1B). The sections were stained with an antibody to rhodopsin and counterstained with the fluorescent nuclear dye diamidino-2-phenylindole dye (DAPI). It is evident that by p14 the FVB/N retina is thinner than normal, has already lost a significant number of rods, and is possibly undergoing a process of phagocytic ablation of rod cell bodies. A further decline can be seen at p21 along with even greater retinal thinning. This leads ultimately to apposition of the retinal pigment epithelium and the surviving neurons, as seen at p28. By the time the FVB/N mice reach p35, the rhodopsin molecule was undetectable in antibody labeling. In contrast, there was robust staining throughout the layer of outer segments at p35 in normal 129 SvEv mice (Fig 1B). It follows that the thickness of the outer nuclear layer (ONL) that contains nuclei of photoreceptor cells is sharply reduced in FVB/N retinas during this period (Fig 1C, left). Clearly, the thickness of the inner nuclear layer (INL), containing the nuclei of bipolar cells (BCs), horizontal cells (HCs), and amacrine cells (ACs), Muller cells, as well as the displaced ganglion cells (GCs) at the inner margin of the INL, seem to be far less affected by the degenerative changes taking place in the distal retina. However, there is a noticeable decline in the thickness of the INL of the FVB/N mice at age of 6 months. These data are shown in Fig 1C, which summarizes the averaged thickness of the ONL and INL measured from the retinal sections obtained from sub-peripheral retinas of the FVB/N and control mice from p14 up to age of 6-months, and each set of data was obtained from four FVB/N and four control animals. In summary, the ONL of the FVB/N retina is significantly thinner at p14 due to rod degeneration, and by p21 the cell content of the ONL consists of a single row of cells, and the thickness of the entire retina has been greatly reduced. Only occasional remnants of rods were visible at p28, and none could be seen by p35, the stage at which rods are possibly ablated by phagocytosis.

**Fig 2:**
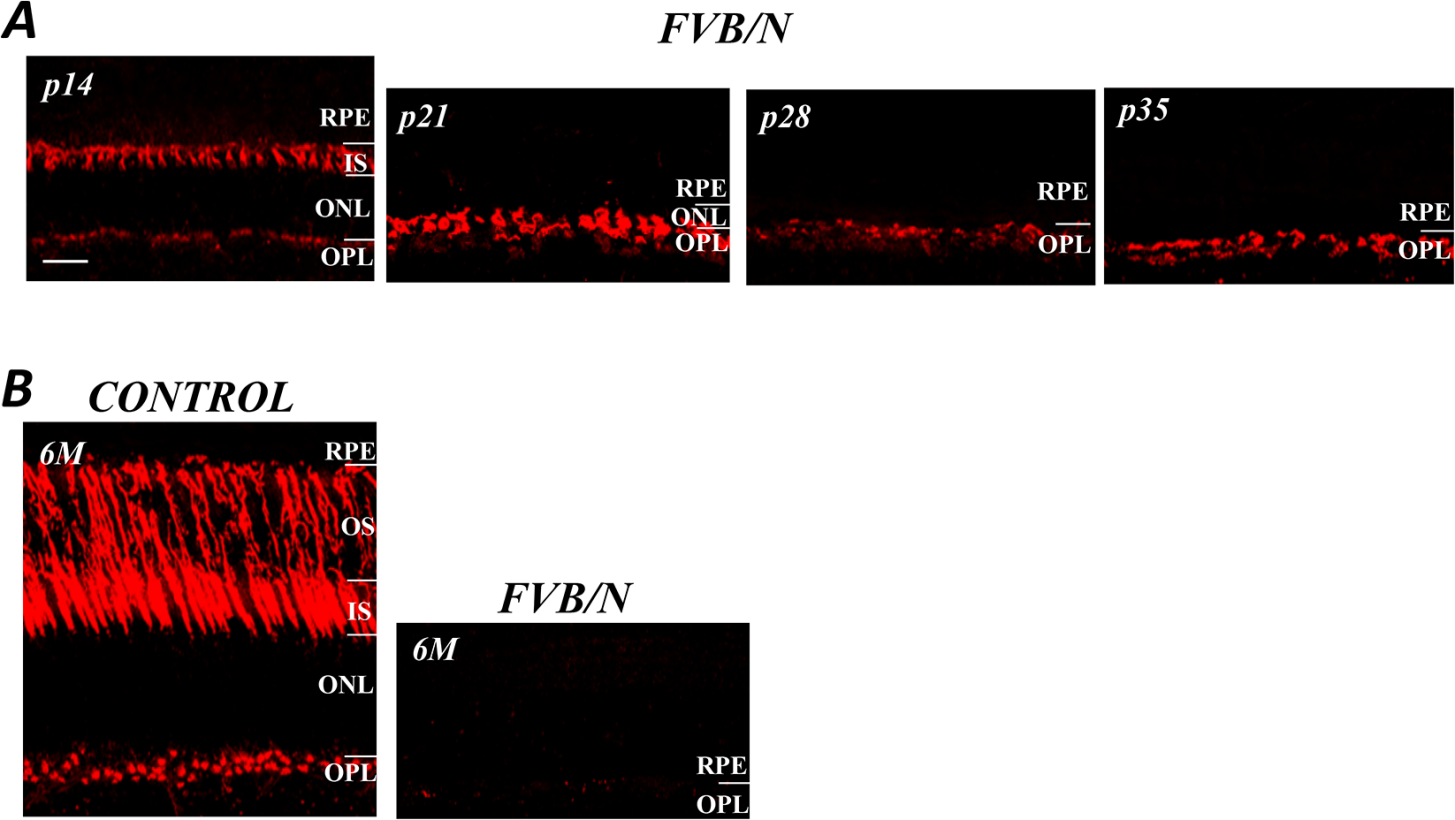
Cone degeneration in the FVB/N mouse. (A) The anti-PNA labeling of cones in the FVB/N mouse distal retina shows their progressive loss and a clear reduction in length. At p35 only the vestiges of some cone pedicles remain. (B) A comparison of the normal cone population with that of the FVB/N mouse at 6-months at which time no cones can be seen. Scale bar, 20μm.

The age-related degeneration of cones in FVB/N retinas was visualized with an antibody against peanut agglutinin (PNA), a lectin that typically labels the matrix sheaths surrounding the outer and inner segments of cones, as well as the cone pedicles [9]. Fig 2 shows the anti-PNA labeled cone photoreceptors in control and FVB/N retinal sections prepared from mice at the ages indicated. As expected, the cones, from their distal to proximal ends, are outlined by the anti-PNA. The images in Fig 2A show anti-PNA labeling from the FVB/N mice at p14, p21, p28 and p34, revealing that there was a significant and progressive reduction in the length and number of cones, at p35 where only the vestiges of some pedicles remain. The images in Fig 2B show the full extent of the anti-PNA labeled cone photoreceptors from the control and FVB/N mouse at 6 months. Labeling was present in the areas of the outer segment (OS), inner segment (IS) and pedicles of cones in the control mouse, whereas in FVB/N retinas there was no PNA-labeling present in FVB/N retinas at 6 months (Fig 2B, right), indicating that all the cones had been lost.

**Fig 3:**
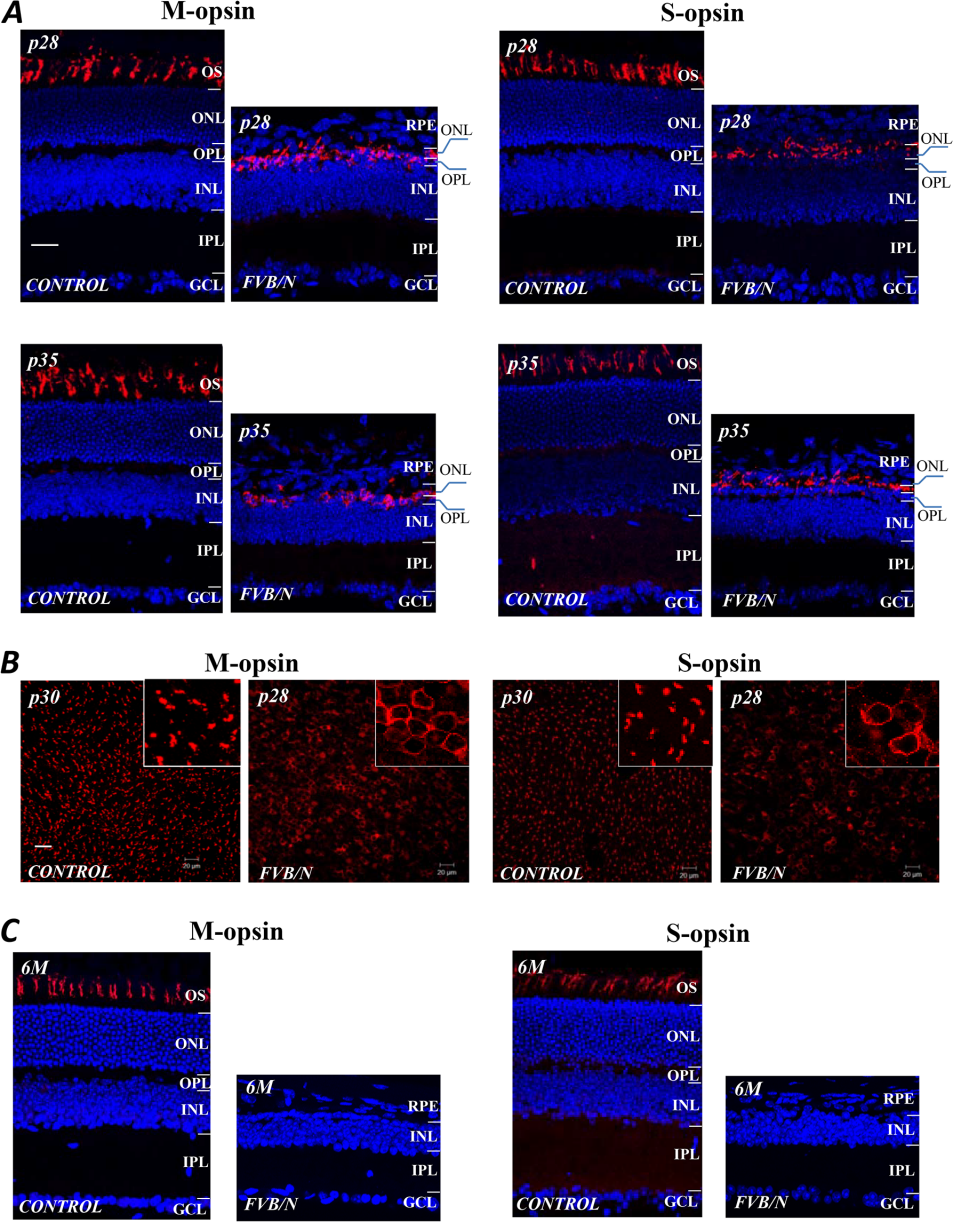
Antibody labeling of M- and S-opsin molecules (red) in the cone OS segments of FVB/N and age-matched controls; the cell nuclei are stained by DAPI (blue). (A) The ONL is almost completely destroyed by p28, and the expression of both opsins appears as single thin line at p35, where the opsin molecules appear to be displaced to the cone somas. (B) confocal images from z-scans of the lamina containing cone OS and nuclei in flat mount preparations at p28 show the translocation of M- and S-opsin molecules to the cone nuclei in the FVB/N retina, compared to its normal location in the cone OS. (C) The absence of antibody labeled opsins in the FVB/N retinal sections from age of 6 months, compared to the age-matched control. OS: outer segment. Scale bars, 20μm.

To investigate further the subtypes of cones and their localizations in normal 129SvEv and FVB/N retinas, we used the antibodies specifically binding to the green and blue-sensitive photopigments, M- and S-opsin; DAPI labeling enabled identification of cell nuclei. The latter shows that the ONL of the FVB/N retina is almost completely destroyed at p28 (Fig 3A). In addition, the expression of both opsin molecules has been reduced, and appears as a single thin line at p35, when the photoreceptors were completely devoid of rhodopsin, which was consistent with the results in Fig 1C. Moreover, there seems to be a translocation of the opsin molecules in the aberrant cones of the FVB/N retina. Fig 3B shows confocal images obtained from z-scans of the lamina containing cone OS and nuclei in flat-mounted retinas prepared from the control and FVB/N mice at the age of p28. They show that M- and S-opsin molecules are present in the cone’s OS in control retinas, whereas in FVB/N retinas the opsin molecules appear now to be displaced to the region of the cone somas (Fig 3B, see insets). Both M- and S-opsin labeling were absent in FVB/N retinas when the mice were 6-months old and all cones were degenerated (Fig 3C).

### The synaptic terminals of photoreceptors

**Fig 4:**
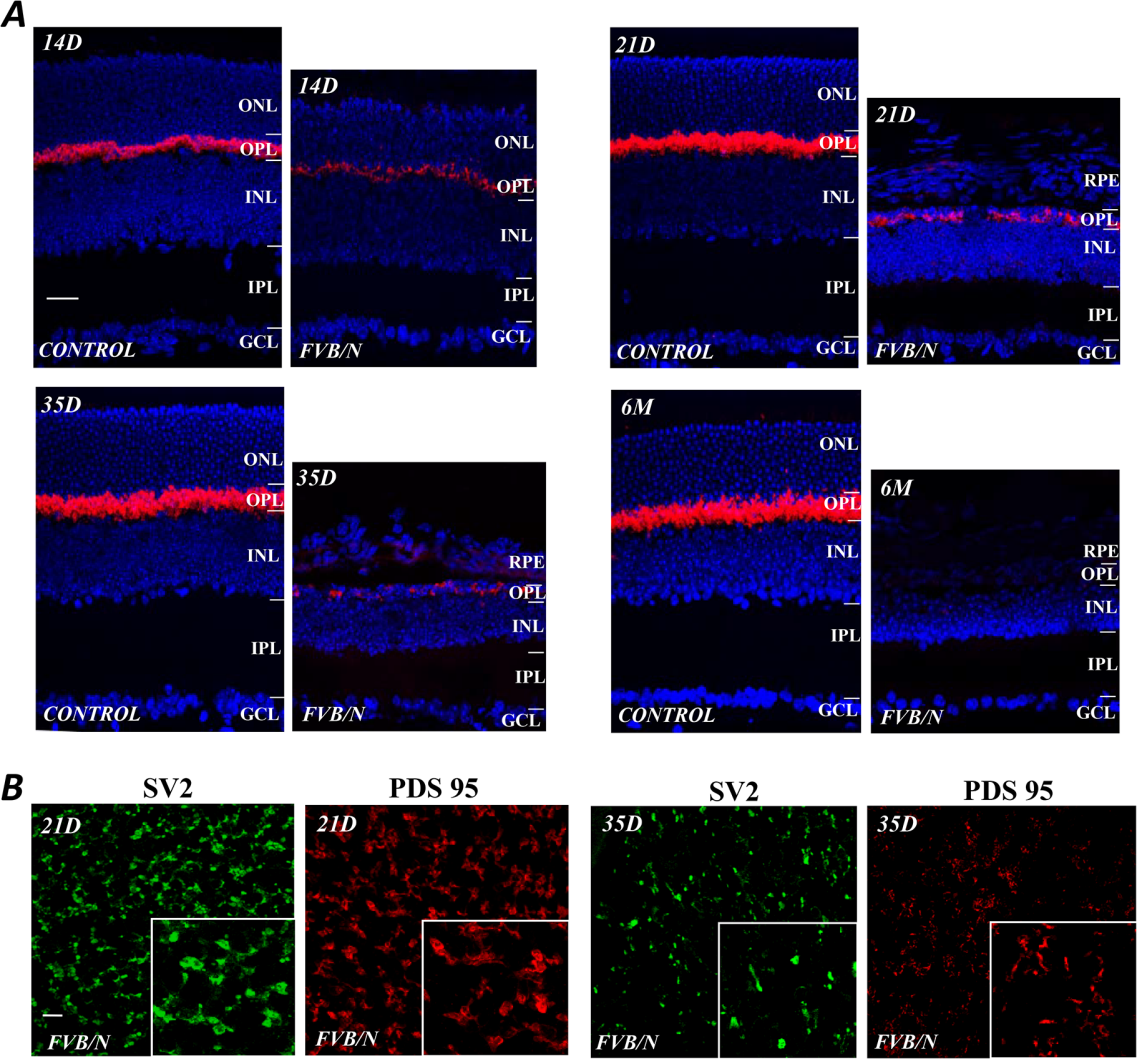
Degeneration of photoreceptor terminals. (A) Anti-PSD-95 labeled photoreceptor synaptic terminals in control and FVB/N retinal sections at various postnatal ages. Note that in the control there was an increased intensity between p14 and p35 in accordance with the age-related increase in visual cells. In contrast, in the FVB/N mouse, PSD-95 labeling was visibly reduced at p14, became progressively weaker at later stages, and was absent at 6 months. (B) Confocal images of anti-SV2 and anti-PSD-95 labeling in flat mount preparations from the FVB/N retinas show a significant amount of labeling present in the OPL at age of p21 and the labeling was reduced at p35. Scale bars, 20μm.

An antibody against the synaptic density protein PSD-95 that is expressed in rod spherules and cone pedicles of mammalian retinas [10] was used to detect changes of the synaptic proteins in photoreceptor terminals. In the control retina, anti-PSD-95 labeling was already present in the photoreceptor terminals at p14, the stage at which mice open their eyes. The labeling gradually intensified up to p35, corresponding to the final stages of photoreceptor maturation in mouse retinas (Fig 4A, CONTROL). In contrast, PSD-95 levels in the photoreceptor terminals of FVB/N retinas were visibly reduced at p14, and a progressively weaker signal was seen in retinal sections at p21and p35; anti-PSD-95 labeling was completely absent in the OPL of the FVB/N retinal sections at 6M (Fig 4A, FVB/N). Note that anti-PSD-95 also produced very weak punctate signals in the IPL of the control and FVB/N retinas throughout the test period, which could be seen in the images with strongly enhanced background (data not shown).

A progressive loss of synaptic proteins in the photoreceptor terminals of FVB/N at age between p21 and p35 was also detected by the antibody to the synaptic vesicle protein 2 (SV2). Fig 4B shows confocal imaging results of anti-SV2 and anti-PSD-95 labeling in the OPL of flat-mounted FVB/N retinas at p21 and p35; it shows a significant amount of labeling present in the retinas at age of p21 and the labeling of the antibodies was reduced at p35 (see insets). Presence of synaptic proteins in the degenerating retinas suggests that some local synaptic circuitry may still exist in the degenerating retinas, but this no longer the case at 6 months.

### The morphology of BCs

**Fig 5:**
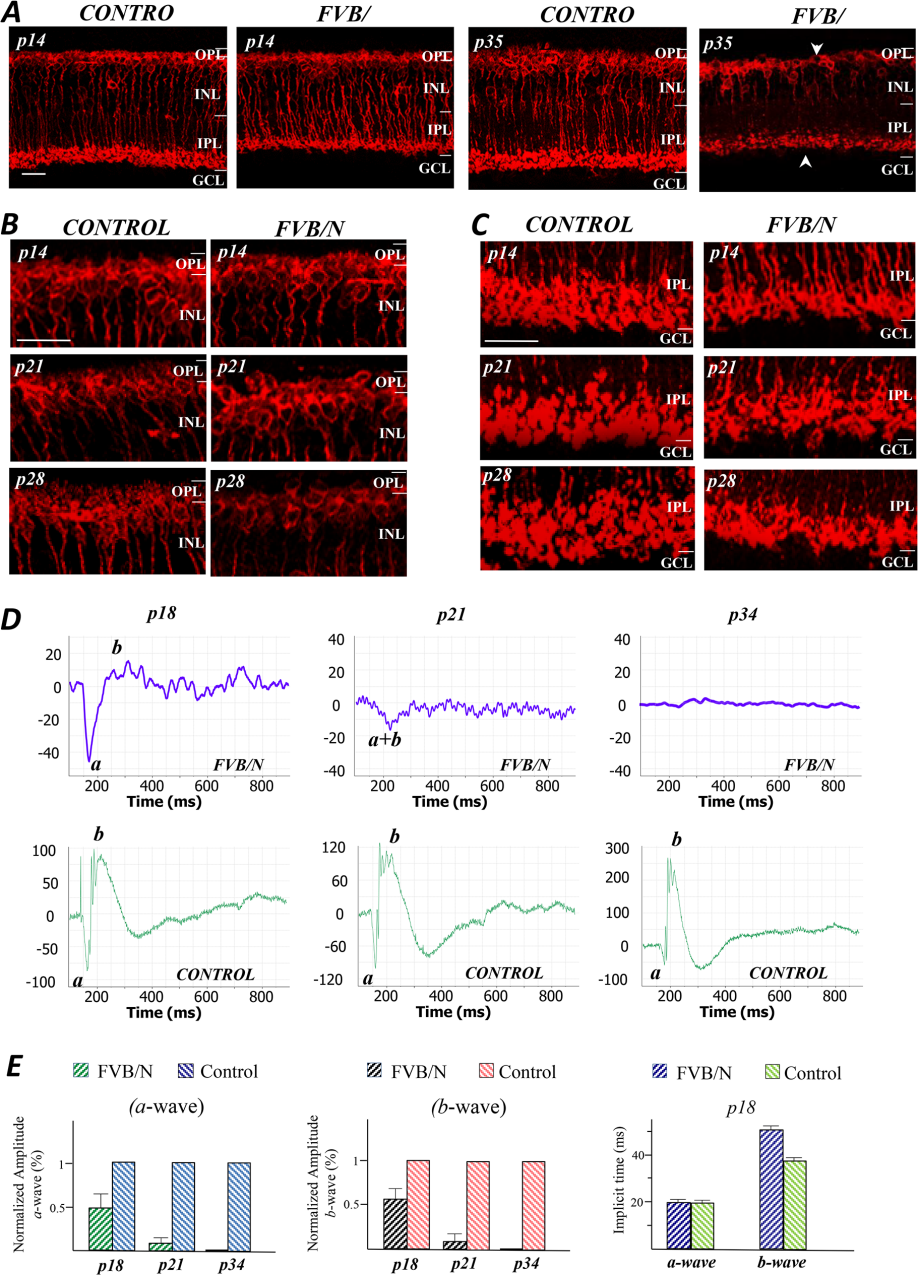
Remodeling of rod-BCs in FVB/N. (A) PKC labeling of rod-BCs shows that their lengths are already reduced at p14, and by p35 there has been a loss of BCs and disruption of the continuity between distal and proximal regions of the cells. (B) Enlarged images of the sections shown in (A) provide evidence of retraction of their dendritic ends at p21 and p28, as well as the progressive destruction of the synaptic terminals (C). (D) ERG recordings from age matched groups of FVB/N and control mice, showed a significant loss of both *a*- and *b*-wave amplitudes in the FVB/N mice at p18, and only a very small long latency *a* wave at p21. By p34, all electrical activity was lost. (E) Bar graphs provide a statistical analysis of the amplitudes and implicit times of *a*- and *b*-wave of ERG data (n = 12) obtained from 3 FVB/N and 3 control mice. Scale bars, 20μm.

To evaluate how BCs react to photoreceptor degeneration in the FVB/N retinas, we used anti-PKC as a cellular marker of rod-BCs. These cells have their dendrites located in the OPL and their axon terminals at the inner border of the IPL. Fig 5A shows labeled rod-BCs in retinal sections of the control and FVB/N at p14 and p35. The lengths of the rod-BCs in FVB/N retinas are already reduced at this stage. By p35 the rod-BCs showed severe disruption between the distal and proximal ends of the cells (arrows). It was difficult to determine whether the dendritic ends of rod-BCs in the FVB/N retina were undergoing retraction at p14, but it was evident in the enlarged images at p21 and p28 (Fig 5B). We also observed atrophy and shrinkage of the synaptic terminals throughout the test period (Fig 5C). Clearly, rod-BCs are severely affected by rod degeneration.

To determine the degree of functional loss, ERG recordings were obtained from FVB/N mice and age-matched controls. The intensity of the light flash generated electrical responses from both rods and cones were recorded from FVB/N and control mice at p18, p21 and p34 (Fig 5D). The two principal components of the light-induced voltage response, the cornea-negative *a*-wave and the positive *b*-wave, derive from the photoreceptors and On-BCs, respectively. For the FVB/N mouse, a reduced amplitude of *a*-wave and small positive *b*-wave response were seen at p18 (note the scale differences in the upper and lower panels in Fig 5D). However, only a long latency *a*-wave remained at p21, and no electrical activity was seen by p34. The amplitudes and implicit times of the *a*- and *b*-wave were also measured from three FVB/N and three age matched control animals (Fig 5E), showing that at p18 the mean (n = 12) amplitudes of *a*-wave and *b*-wave of FVB/N mice were respectively reduced by 50±16% and 38±10% of the control; by 86±6% and 84±8% of the control at p21. As already indicated, no consistent measurable signals were obtained at p34. The differences in implicit times at p18 were nearly equivalent, i.e., the average value for the *a*-wave measured from FVB/N and control were 22±6 ms and 20±6 ms, respectively; however, the mean value of implicit times for the *b*-wave were 52±7 ms and 38±4 ms, respectively. Thus, the early and profound decline in the ERG reflects the severe destruction of photoreceptors and On-BCs.

**Fig 6:**
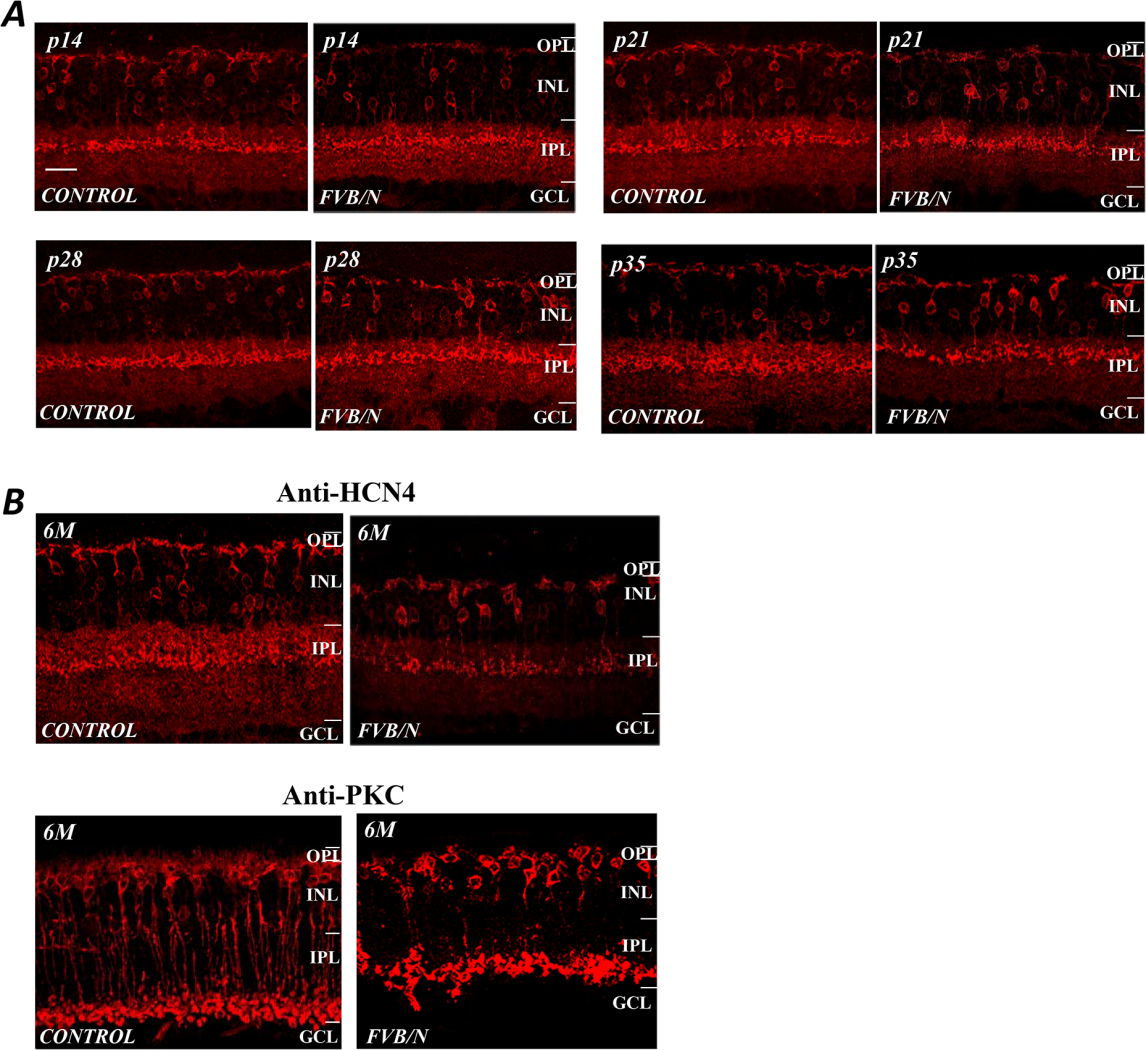
Remodeling of the cone-Off-BCs. (A) The structure of the cone-driven Off-BCs as revealed by the HCN4 antibody shows that these cells appear to be relatively unaffected by the photoreceptor degeneration at p14 and p21. Nevertheless, by p35 there are abnormal structural changes. (B) By 6 months their numbers had been reduced and their morphology had been disrupted, but to a lesser extent than those of rod-BCs labeled by anti-PKC. Scale bar, 20μm.

The results from anti-HCN4 labeling of the cone-driven Off-BCs show that photoreceptor degeneration has only a modest effect on the morphology of the cone-driven Off-BCs at ages of p14 to p35 (Fig 6A) when the structures of the rod-BC are severally altered (see Fig 5A), and it is known that anti-HCN4 labels the type 3a Off-BCs that make synapses with cone terminals [11]. However, note that by 6 months the BCs morphology in FVB/N retinas had undergone disruptive changes and the anti-HCN4 labeling was reduced (Fig 6B). Although the dendritic morphology of the Off-BCs appeared altered in the FVB/N retinas at 6-month, the structure of the axon terminals of the cells seemed to be intact, which was in contrast to the appearance of rod-On-BCs labeled by anti-PKC (see Fig 6B).

### The morphology of horizontal cells (HCs)

**Fig 7:**
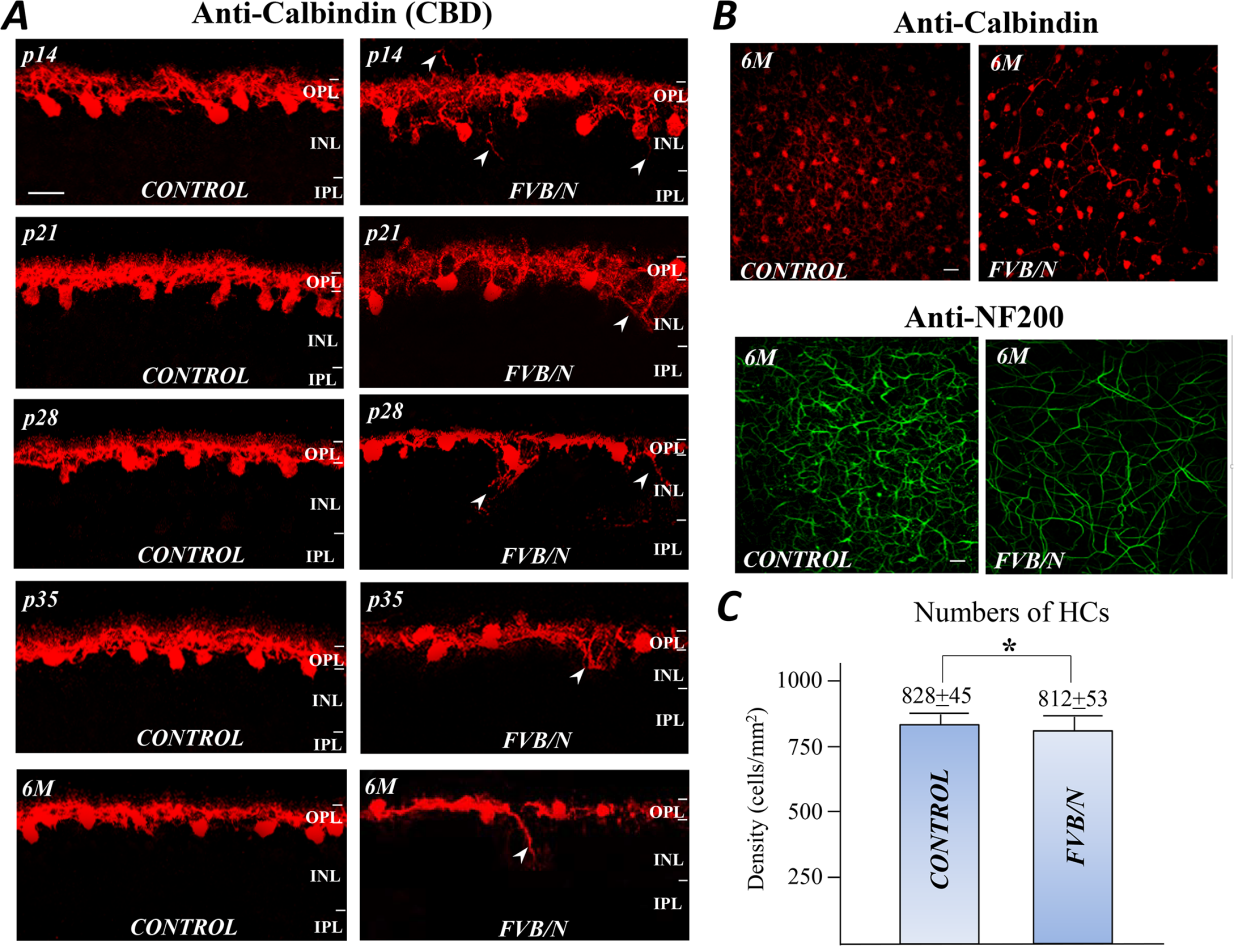
Remodeling of HCs. (A) The anti-calbindin labeled HCs from control and FVB/N retinal sections at various ages show that as early as p14, HCs sprout new processes that extend proximally and distally to neighboring cells (see arrow heads) during photoreceptor degeneration. The distal processes tend to retract but those approaching the BC layer continue to sprout for up to 6 months of age. (B) The anti-calbindin and anti-NF200 labeled HC axons in the control and FVB/N retinal flat-mounts. (C) The numbers of calbindin-positive HCs counted from 6 retinas of the control and 6 retinas of the FVB/N. Scale bars, 20μm. * p >0.05.

HCs are interneurons that modulate the synaptic information between photoreceptors and BCs in the OPL. Their processes extend distally to contact with photoreceptors; whereas gap junctions between neighboring HCs form an electrically coupled syncytium that broadens its receptive field. The somas and processes of HCs can be visualized with the antibody against the calcium binding protein calbindin-D28 [12]. As shown in Fig 7A, strong labeling was found in HC processes in the OPL, and in the cell somas located at the outer margin of the INL. Although both control and FVB/N retinal sections exhibited similar patterns of labeling, the retinal sections from FVB/N mice show that at p14 several sprouting processes appeared and extended into the distal and proximal retinal layers (arrows). From p21 to 6 months of age, during which time the HCs of the control retinas showed only dendrites extending to the synaptic terminals of the visual cells (Fig 7A, CONTROL), the HCs of the FVB/N retina continued to sprout new processes into the inner retina, and retract their distal processes. These results indicate that HCs in the FVB/N start a process of neuritogenesis as early as at p14; the process continues and leads to microneuromas throughout the stages of the retinal degeneration.

The remodeling of the axonal processes of HCs in FVB/N was detected by the antibody against the neurofilament 200 (anti-NF200), a cellular marker for mouse HC’s axons [13]. Fig 7B shows confocal images of anti-calbindin-D28 and anti-NF200 labeled HC axons in flat mount retinas from control and FVB/N mice at age of 6 months. It shows that the density of HC axonal processes in FVB/N retinas was visibly reduced compared to the age-matched control. Note also that HC somas were evenly distributed in the control retina, but they appeared to be clustered in FVB/N retina (Fig 7B, top panel). The numbers of calbindin-D28 positive HCs were counted in the middle and peripheral areas of the control and FVB/N retinas at age of 6 months. As shown in Fig 7C, the average cell numbers obtained from 6 eyes in each preparation indicate that there are 828±45 (mean±SEM) cells per mm^2^ from the control retinas and 812±53 cells per mm^2^ from the FVB/N retinas (*p* >0.05, n = 6). The results suggest that there is no significant difference between these two preparations. Possibly, the density of HCs in the FVB/N retina is unaffected by the massive photoreceptor degeneration.

### The third-order neurons in FVB/N retina

**Fig 8:**
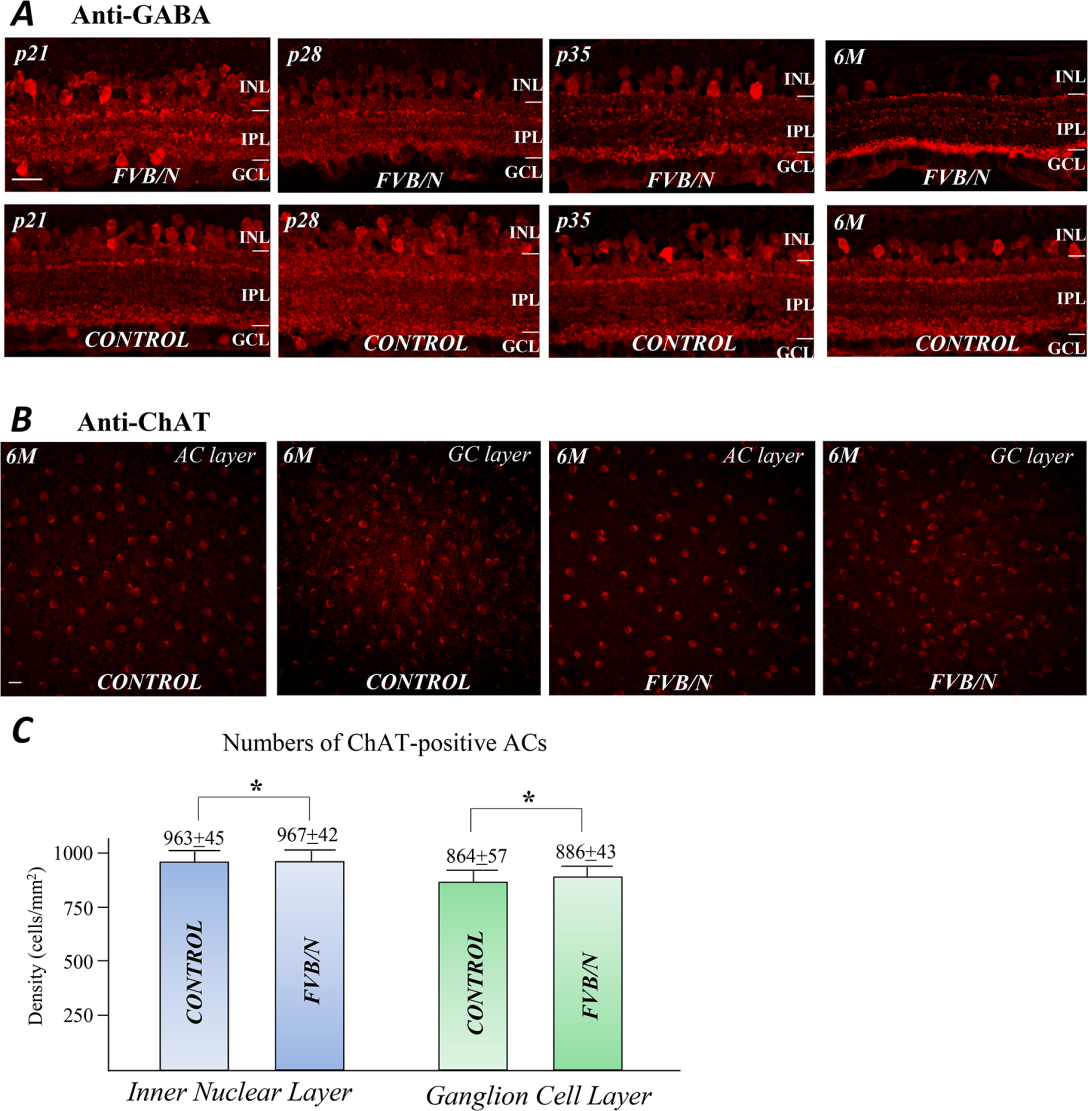
ACs in the control and FVB/N retinas. (A) Labeling of GABA immunoreactive ACs reveals the progressive thinning of the INL during the course of the disease process in the FVB/N retina. (B) Anti-ChAT labeling of starburst ACs in flat-mounted retinas prepared from the control and FVB/N mouse at age of 6 months. (C) The average members of ChAT-positive ACs counted from the INL and GCL in the control and FVB/N retinas. Scale bars, 20μm. * p >0.05.

To determine whether ACs and GCs are affected by photoreceptor degeneration in FVB/N retina, the GABA immunoreactive ACs were analyzed in the control and FVB/N retinas at ages of p21, p28, p35 and 6-month. Fig 8A shows an example of anti-GABA labeled ACs in both preparations. There was no visible structural change in the GABAergic ACs at p21, despite the extensive photoreceptor degeneration. However, at later stages there was a progressive thinning of the IPL. By age of 6 months most of the GABA-immunoreactive ACs still survived, although the intensities of GABA labeling were reduced in some of the GABAergic ACs.

To determine whether a group of direction-selective ACs would survive in the degenerated retina, the specific antibody against choline acetyltransferase (anti-ChAT) was used to mark the ChAT-positive ACs that are known as starburst ACs and that are located in both INL and GC layer (GCL). Fig 8B shows sample images of ChAT-positive ACs in the INL and GCL, taken from flat-mounted retinas of the control and FVB/N at age of 6 months. The densities of the ChAT-positive ACs in the control and FVB/N appeared to be the same. The numbers of ChAT- positive ACs were counted and compared at the INL and GCL in the flat-mount retinas from the two preparations. As the results showing in Fig 8C there are ±45 and 967±42 cells per mm^2^ (*p* >0.05, n = 6) respectively in the INL of the control and FVB/N retinas; correspondingly, there are 864±57 and 886±43 cells per mm^2^ (*p* >0.05, n = 6) in the GCL of the control (n = 6) and FVB/N retinas (n = 6). The numbers of ChAT-positive ACs are slightly reduced in the FVB/N retina at 6 month.

**Fig 9:**
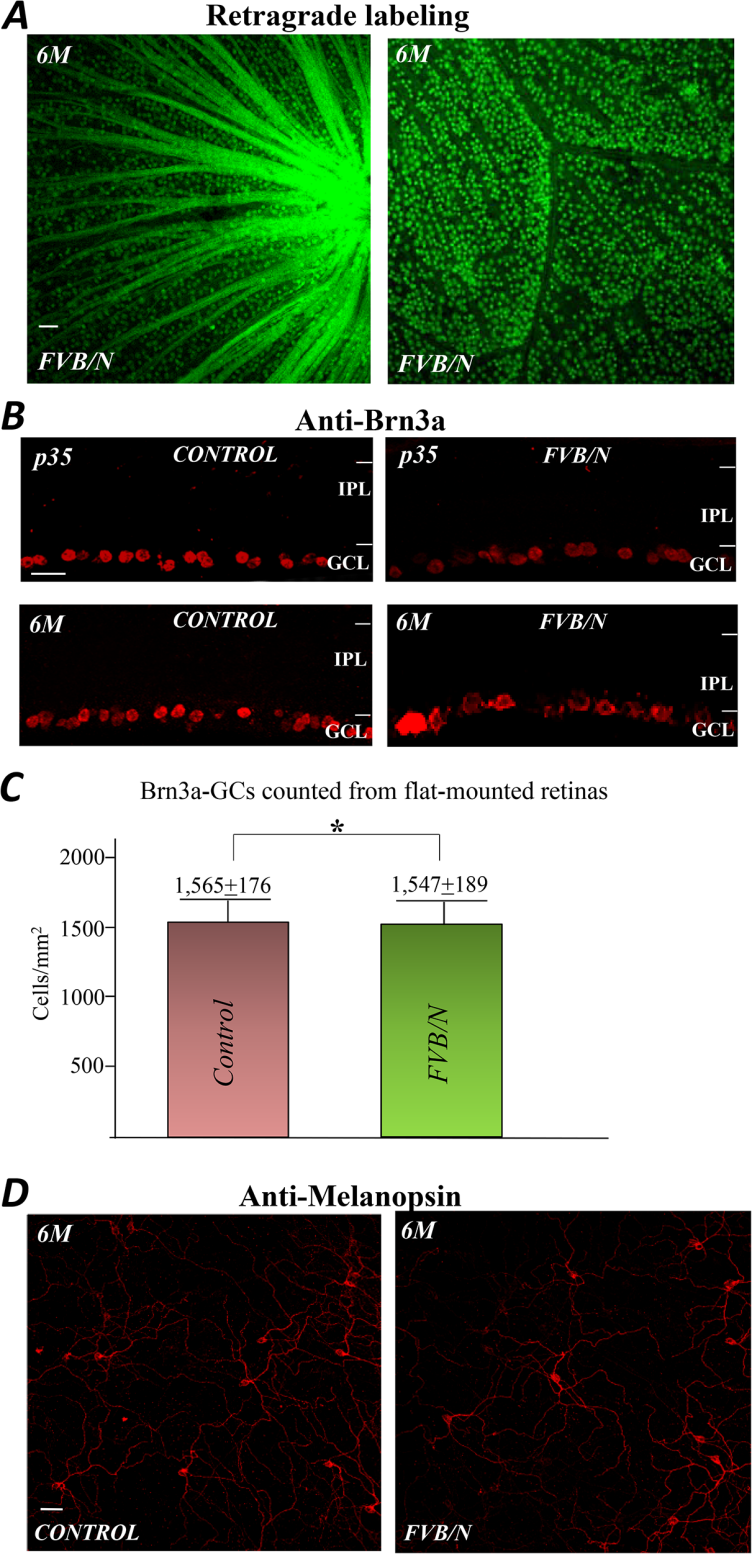
Immunolabeling of retinal GCs in FVB/N and control mouse. (A) Lucifer Yellow retrograde labeling of the optic nerve and GCs in a flat mounted preparation of the FVB/N retina at 6 months of age shows a normal optic nerve fiber pattern and retention of a substantial number of GCs. (B) The sections labeled with anti-Brn3, a GC marker, in control and FVB/N at ages of p35 and 6-month show only minor differences in number and location. (C) The numbers of GCs counted from flat-mounted retinas prepared from the control and FVB/N at age of 6-months. (D) Melanopsin positive GCs in control and FVB/N retinas (6-months) appear quite similar in number and location. Scale bars, 20μm. * p >0.05.

Lucifer Yellow retrograde labeling revealed that optic nerve fibers appeared normal in FVB/N retinas at 6-month (Fig 9A, left) and a substantial number of GCs were still present in the retinas (Fig 9A, right). To determine whether the density of GCs is affected by loss of photoreceptors in the FVB/N, the antibody against Brn3a, a transcription factor that expresses in the GCs, was used to label the GCs in the FVB/N retinas and the age matched control. Fig 9B shows the anti-Brn3a labeled GCs in retinal sections at ages of p35 and 6-months. The numbers of Brn3a-positive GCs were counted from the two preparations in flat-mounted tissues. Fig 9C shows the cell counting results for the GCs from 8 retinas, 4 for each preparation, indicating that the mean values of Brn3a-positive GCs in the control and FVB/N retinas (6-month age) are 1,565±176 cells per mm^2^ and 1,547±189 cells per mm^2^ (*p* >0.05, n = 4), respectively. A comparison suggests that there were no marked differences on the numbers of GCs between the two preparations.

There is a special group of GCs that expresses melanopsin, a light sensitive molecule implicated in the regulation of circadian rhythms and control of the pupillary light response [14–15]. The melanopsin expressing GCs, known as intrinsically photosensitive retinal GCs (ipRGCs), can be visualized with an antibody against this protein. Fig 9D shows anti-melanopsin labeled ipRGCs in flat-mount retinas from the control and FVB/N at 6 months. Here too the densities of the ipRGCs in the two preparations appear to be the same, consistent with the results in previous studies that ipRGCs are relatively unaffected by photoreceptor degeneration (cf. Zaidi et al., 2007) [16]. The existence of ipRGCs in FVB/N mice suggests that the FVB/N mice still possess a physiological response to changes in ambient light, photoentrainment, a pupillary light reflex, etc., related aspects of visual functions.

### Other pathological reactions

**Fig 10:**
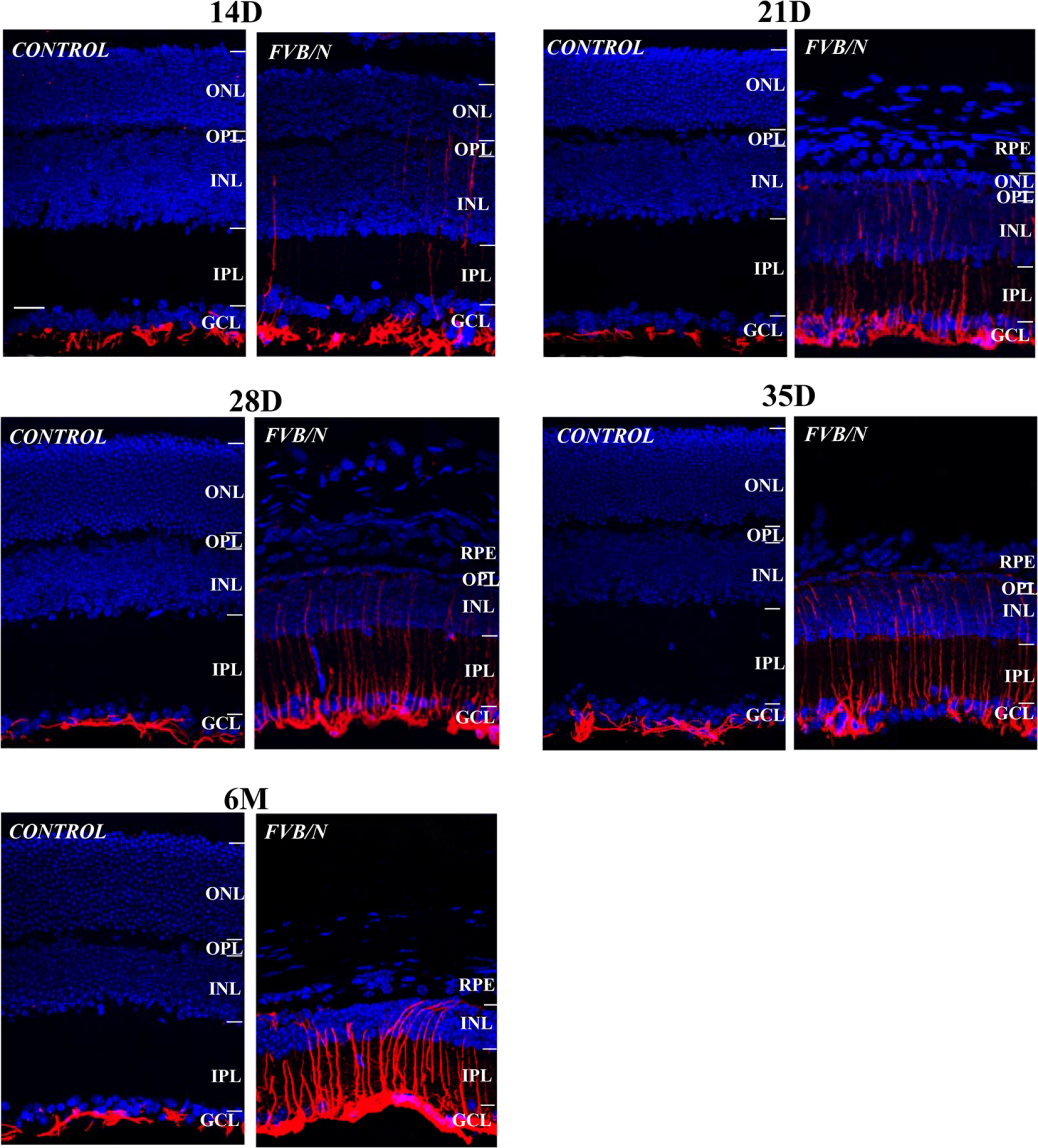
A comparison of anti-GFAP labeling in control and FVB/N retinal sections. It shows that in the normal retina GFAP is present in astrocytes at the innermost margin of the retina, a pattern that continues unchanged through adulthood. In contrast, the FVB/N retina displays a stronger GFAP labeling at p14; expression progressively increases from p21 to adulthood, as the marker begins to climb the length of the Muller cells, progressing from the endfeet to the INL. Scale bar, 20μm.

Glial fibrillary acidic protein (GFAP) is the main intermediate filament protein in mature astrocytes [17], but it is often expressed in Muller cells in response to retinal injuries and the degenerative changes associated with genetically-mediated retinal dystrophy [18]. We used anti-GFAP as a biomarker to detect its presence in the FVB/N retina during photoreceptor degeneration. Fig 10 shows that in the normal retina GFAP labeling is present in astrocytes at the innermost margin of the retina where it is in close apposition to GCs and there is no change throughout the 6 month study. In contrast, the FVB/N mouse retina displays a more intense labeling at p14, and GFAP expression is readily seen to be in Muller cells at p21. Moreover, there is a progressive increase in GFAP expression at later stages when it begins to climb the length of the Muller cells, progressing from the endfeet to the INL. There is no sign of similar changes in the age-matched control retinas.

## Discussion

This study provides for the first time a detailed description of the progressive loss of cellular structure in the FVB/N retina, a mouse model of severe, early-onset retinal degeneration. Although closely related in genotype and phenotype to the *rd1* mouse, which has been studied extensively [19–22], this strain was developed independently in the early 1970s and the genetic background of this strain was well-characterized by Taketo et al. (1991) [6]. In the current study, we used immunocytochemical and electrophysiological methods to examine the structural changes that occur at every cell type in the FVB/N retina, its functional consequences, and some aspects of neural remodeling.

Since FVB/N mice have a genetic defect of the *pde6b* gene that encodes the β-subunit of PDE, there is rapid photoreceptor degeneration during postnatal retinal development, and soon thereafter, total loss of visual response in the third weeks after the birth. These are accompanied by structural changes of the OS and ONL. The ONL was visibly thinned at p21 due to loss of rods and the remaining cones displayed pathological changes, including shortening of their OS. Indeed, most cones lose their OS at this age (Fig 2A), and by 6 months all cones had degenerated (Figs 2B and 3C). The rapid rate of photoreceptor degeneration in FVB/N retina is consistent with the findings from the *rd1* mouse [23]. As the *rd1* mouse studies show that cones display morphological abnormalities at p8 when photoreceptors are in early stages of synaptogenesis [24–25], we assume that a similar process may have occurred in the cones of FVB/N. In addition to the *rd1* mouse, there is another mouse model for recessive retinal degeneration that also carries *pde6b* gene mutation called *rd10*. The differences existing in the genetic mutations of these to mouse model are that the *rd10* mouse carries a missense mutation (R560C) in exon 13 of the *pde6b* gene, whereas *rd1* mutation is caused by an insertion of murine leukemia provirus near the first exon combined with a point mutation which introduces a stop codon in exon 7. As a result, the *rd10* mouse shows a later onset and slower rate of retinal degeneration; usually, the rods in the *rd10* mouse start to degenerate at age of p16 and all rods are lost by 2 months [23]. It has also been reported that ERG recording form the *rd10* is never abnormal [13].

ERG recordings from FVB/N mice show that there are *b*-wave responses from the mice at p18, evidencing that cones are still able to make synapses with the On-BCs, although the synapses might be strongly affected by rod degeneration. The *b*-wave response in the FVB/N retinas was diminished at p21 (see Fig 5D). However, the antibody-labeling results reveal that a substantial amount of the synaptic proteins: PSD-95 and SV2, are still present in cone axon terminals when the structure of cones are significantly altered at p28 (see Fig 4B). Hence, the presence of the synaptic proteins in the cone pedicels lasts even to the age of p35 when the rods are ablated by phagocytosis in the FVB/N retinas. However, cones are possibly unable to conduct phototransduction cascades since cones undergo pathological alterations, such as loss of their OS and dislocation of opsin molecules (see Fig 3B). This may be a reason for the loss of light-evoked *a*- and *b*-wave responses in the FVB/N in the early stages of retinal degeneration. Whether cone terminals filled with synaptic proteins are able to make local miniature glutamate synapses with the second-order neurons remains undetermined. We speculate that such local synapses from the remnant cones may contribute to neuronal survival and remodeling processes of the BCs, including HCN4-positive Off- BCs in the degenerative retina. Although it suggests that some remnants of local synaptic circuitry might still exist in the distal retinas of FVB/N at p35, this may be no longer the case at 6 months when both rods and cones are absent from the retinas.

We noticed that HCs displayed neurite sprouting at postnatal day 14 and exhibited considerable changes of morphology after p21 when the branches of HC’s process are anomalously descending to the IPL, undergoing microneuroma formation, suggesting that HCs might switch their synaptic targets. These results are consistent with an early study of neuritogenesis of HCs in the developing FVB/N retina [26]. Similar sprouting of HC processes has also been noted in Royal College of Surgeons rats [27], in adult cat retinas after experimental detachment [28–29], in human retinas with retinitis pigmentosa [2], and in retinas of *rd1* mice [3]. However, there is no convincing evidence that functional synapses are formed with any of the cells they contact. Despite these structural changes, many of the HCs in the FVB/N retina survived throughout the 6-month test period (cf. Fig 8C). Although the lines of evidence suggest that the calcium-sensitive kinase βCaMKII and retinoic acid promote neuritogenesis in the degenerate retinas [30–32], the mechanism(s) responsible for the sprouting of HCs into the IPL remain unclear. A study suggests that calbindin, an intracellular Ca^2+^ binding protein, in HCs may play a role in control of intracellular Ca^2+^ levels that initiate neurite sprouting in HCs in the situation of abnormal glutamate released from damaged photoreceptors [33].

It seems that many of the third-order neurons are spared from neurodegeneration despite the rapid and massive photoreceptor degeneration. Although most BCs have remodeled extensively and also abnormal glutamate synapses occur in the IPL, there is no detectable change in cell densities of the GABA- or ChAT-reactive ACs at 6-month old FVB/N; a this age virtually no functional photoreceptors are left in the retina. Although the current study demonstrates no significant changes in the densities of the ACs in the FVB/N retina, we are not ruling out the possibility that any microstructural change occurs in the projection arbors of the ACs in the FVB/N at age of 6 months. The early studies from the *rd1* mouse show that there are morphological changes, such as 10–50% reduction of dendritic field size in subclasses of GCs [34–35]. But another study reported no changes of GC’s morphology are also reported in the *rd1* (cf. Lin and Peng, 2013) [36]. Our results are consist with the notion that photoreceptor degeneration has minimum effect on the inner retinal neurons in FVB/N mice. Since our study did not examine the changes of receptive field and dendritic field size of ACs and GCs, we can’t rule out the possibility that remodeling occurred in the dendrites of the ACs and GCs in the FVB/N retina in the late stages of photoreceptor degeneration.

## Conclusions

This paper contains the first detailed study of the neurodegeneration in FVB/N mouse retina. The distal neurons and Muller cells undergo a progressive form of structural change. The resultant structural changes are typically referred to as remodeling [37–38], the term inferring that there are some abnormal neural connections generated in pathological and diseased retinas. However, it is largely unknown whether substantial cellular remodeling in pathological and disease conditions is a detrimental or beneficial process for cell survival or remaining physiological functions. In the FVB/N retina, we found no structural change that could be interpreted as being beneficial. Nevertheless, the FVB/N strain may serve as a potentially valuable model for studying the cellular and molecular mechanisms of cell degeneration, survival, and remodeling in retinal pathological conditions. In addition, some of the unique characteristics of the inbred FVB/N mouse are its vigorous reproductive performance and consistently large litters, which are beneficial for genetic, molecular, and histological studies of photoreceptor degenerative diseases.
